# 18F-FDG-PET/CT for polymyalgia rheumatica: agreement and diagnostic accuracy of routine PET scan report vs. standardized PMR PET scores

**DOI:** 10.3389/fnume.2025.1550881

**Published:** 2025-03-10

**Authors:** Kornelis S. M. van der Geest, Rob G. J. Grootelaar, Karin Bouwman, Maria Sandovici, Andor W. J. M. Glaudemans, Elisabeth Brouwer, Riemer H. J. A. Slart

**Affiliations:** ^1^ Department of Rheumatology and Clinical Immunology, University of Groningen, University Medical Center Groningen, Groningen, Netherlands; ^2^ Department of Nuclear Medicine and Molecular Imaging, University of Groningen, University Medical Center Groningen, Groningen, Netherlands; ^3^ Department of Biomedical Photonic Imaging, Faculty of Science and Technology, University of Twente, Enschede, Netherlands

**Keywords:** FDG-PET/CT, polymyalgia rheumatica, scores, scan report, treatment, sensitivity, specificity, agreement

## Abstract

**Background:**

^18^F-FDG-PET/CT may reveal widespread inflammation of musculoskeletal structures in polymyalgia rheumatica (PMR). Currently, scans are subjectively analysed based on the overall gestalt of the scan. Standardized PET scores may potentially aid the interpretation of the scans for suspected PMR. Here, we compared the agreement and diagnostic accuracy of routine PET scan reports vs. the most validated PET scores for PMR.

**Methods:**

68 consecutive patients with suspected PMR (treatment-naïve, *n* = 29; already treated, *n* = 39) undergoing ^18^F-FDG-PET/CT were included. In glucocorticoid-treated patients, complete tapering was pursued prior to the scan. Conclusions of routine PET scan reports were interpretated by three independent readers as “PMR”, “not PMR” or “unclear”. The Leuven and Leuven/Groningen scores were determined. Agreement of scan report interpretation, and agreement of routine scan reports and PET scores were determined. Sensitivity and specificity were determined for the routine scan report and the two scores, with the clinical diagnosis established after 6 months follow-up as the reference standard.

**Results:**

A diagnosis of PMR was made in 45/68 patients. Routine scan reports were uniformly rated by all three readers in 54 (78%) cases. Following a consensus meeting, scans were rated as “PMR” in 43 cases, “unclear” in 10 cases and “not PMR” in 15 cases. The routine scan report showed a sensitivity of 82% and specificity of 74%, if “unclear” cases were considered negative for PMR. The Leuven and Leuven/Groningen Scores showed similar diagnostic accuracy. Agreement between the routine scan report and PET scores was good (Cohen's kappa 0.60–0.64), if “unclear” cases were excluded from the analysis. Among 8/10 “unclear” cases, the PMR PET Scores accurately distinguished between PMR/PMR-mimicking inflammatory conditions and non-inflammatory conditions. Agreement and diagnostic accuracy of routine scan reports and PET scores were better among treatment-naïve patients than those that had been treated previously.

**Conclusion:**

Our study reveals that routine PET scan reports for suspected PMR can be interpreted differently between readers. Although the routine PET scan reports and PMR PET scores did not always agree, they demonstrated similar diagnostic accuracy, with the highest accuracy observed in treatment-naive patients. The Leuven and Leuven/Groningen score could especially be helpful for cases in which the nuclear medicine physician is uncertain.

## 1 Introduction

Polymyalgia rheumatica (PMR) is a prevalent rheumatic inflammatory disease in the elderly. PMR causes severe pain and stiffness of the shoulders and hips, often with high inflammation markers in blood. Imaging studies have shown that PMR is characterized by widespread inflammation affecting the bursae, tendons, and joints. PMR is considered part of a single disease spectrum with giant cell arteritis, an inflammatory condition affecting large and medium arteries (1). Glucocorticoid treatment remains the cornerstone of treatment in PMR, but targeted therapies are now emerging for this condition (2–4).

Many clinicians diagnose PMR based on the presenting symptoms and laboratory findings, but imaging tests can be helpful in confirming the diagnosis in atypical cases, or to rule out alternative, serious conditions. Ultrasonography and magnetic resonance imaging may reveal musculoskeletal inflammation in specific areas, such as the shoulder and/or hip girdle (5). ^18^F-FDG-PET/CT is the only whole body imaging method for PMR, and reveals widespread distribution of inflammatory lesions in patients with PMR (6). A meta-analysis reported a sensitivity of 85% and specificity of 80% of ^18^F-FDG-PET/CT for a diagnosis of PMR (7). ^18^F-FDG-PET/CT is also applied when clinicians seek to rule out giant cell arteritis or other critical conditions such as cancer or infections. Despite its high cost, ^18^F-FDG-PET/CT may thus be a helpful diagnostic tool in selected cases to further confirm or rule out a diagnosis of PMR.

To date, the evaluation of ^18^F-FDG-PET/CT scans for suspected PMR remains a subjective process. Routine PET scan reports are based on the interpretation by individual nuclear medicine physicians. Such interpretation may be guided by the overall “gestalt” as well as specific patterns of metabolic uptake the reader might expect in case of PMR. When performed by experts with a specific interest in PMR, high sensitivity and specificity are obtained by such interpretation (8). However, it is unclear how accurate scan reports are in daily clinical practice, when performed by readers with less expertise in PMR. If the reports' conclusions are ambiguous, this might add further subjectivity related to the reports' interpretation by the treating clinicians requesting the scan. This could potentially influence important treatment decisions for individual patients.

In order to standardize the interpretation and reporting of ^18^F-FDG-PET/CT results for suspected PMR, various “PMR PET scores' and algorithms have been introduced (9–12). To date, the Leuven Score and Leuven/Groningen Score have shown the highest and most consistent diagnostic accuracy in different patient cohorts (9, 10, 13, 14), albeit with limited ability to discriminate between PMR and other rheumatic inflammatory diseases affecting the shoulders and hips (8, 10). Both PMR PET scores rely on visual grading of ^18^F-FDG uptake at predefined anatomic sites: 12 sites for the Leuven Score and 7 sites for the Leuven/Groningen Score (9, 10). It is currently unclear whether these PMR PET Scores might be of additional benefit when compared to results reported by nuclear medicine physicians in daily clinical practice.

In the current study, we compared the conclusions of routine ^18^F-FDG-PET/CT scan reports of scans performed for suspected PMR with the aforementioned objective PMR PET scores in terms of agreement and diagnostic accuracy. Furthermore, we evaluated whether conclusions of routine PET scan reports were clear and uniformly interpreted by different readers.

## 2 Materials and methods

### 2.1 Patients

This is a retrospective study of consecutive patients referred for suspected PMR to the Department of Rheumatology and Clinical Immunology at the University Medical Center Groningen from December 2018 to May 2022, and who underwent ^18^F-FDG-PET/CT. This included treatment-naïve patients with suspected new-onset PMR, as well as patients that were previously diagnosed with PMR but in whom diagnostic doubt arose later. If patients were still using glucocorticoid treatment at referral, complete tapering of glucocorticoid treatment was pursued prior to ^18^F-FDG-PET/CT. In case patients were unable to completely taper the treatment due to severity of symptoms, a maximum prednisolone dose of 5 mg was allowed during the scan. Patients with concomitant giant cell arteritis were excluded. The study protocol was reviewed by the Medical Ethical Committee of the UMCG (#202100360) and given the retrospective nature of the study no informed consent was required.

### 2.2 Reference standard

The final clinical diagnosis, as established by an expert rheumatologist (KvdG, MS, EB) after 6 month of follow-up, was used as the reference standard for PMR. The diagnosis was based on the complete history, physical examination, laboratory investigation and other imaging tests (e.g., musculoskeletal ultrasonography). The clinical experts were aware of the routine PET scan report of the ^18^F-FDG-PET/CT scan, but not of the outcomes of the objective ^18^F-FDG-PET/CT scores for PMR.

### 2.3 ^18^F-FDG-PET/CT procedure

Blood glucose was examined 1 h before the ^18^F-FDG injection and had to be below 10.8 mmol/L, as we previously found similar PMR PET scores in PMR patients with serum glucose <7 mmol/L and those that had glucose levels 7.0–10.8 mmol/L (10). All scans were performed using a Biograph mCT 40 or 64-slice PET/CT or Biograph Vision PET/CT with 3 min per bed position or a Biograph Vision Quadra PET/CT with 5 min scan of the entire long axial field of view (all camera systems: Siemens Healthineers, Knoxville, TN, USA). Patients were scanned from the vertex of the skull up to the knees. Patients fasted for a minimum of 6 h before 2 or 3 MBq (depending on the used camera system) i.v. ^18^F-FDG/kg body weight was administered. Scan acquisition was performed 60 min after i.v. ^18^F-FDG administration. Low-dose CT was performed for attenuation correction and anatomic mapping with 100 kV and 30 mAs.

### 2.4 Analysis of routine PET scan report

The conclusions of the routine PET scan reports were collected and independently rated by three readers that were blinded to the clinical data (KvdG, RG and RS) as “PMR”, “not PMR” or “unclear”. Thereafter, cases of discrepancy between readers were resolved in a consensus meeting held between the three readers.

### 2.5 PMR PET scores

Visual assessment of ^18^F-FDG uptake at specific musculoskeletal sites was performed by an expert nuclear medicine physician (RS), who was blinded to the clinical data. ^18^F-FDG uptake was graded as follows: 0 = no uptake, 1 = uptake lower than liver, 2 = uptake similar or higher than liver. The following sites were examined to calculate the Leuven Score (9): the cervical and lumbar interspinous bursae, bilateral sternoclavicular joints, ischial tuberosities, greater trochanters, hips and shoulders (*n* = 12 sites, max score 24, cut-off 16). Furthermore, the Leuven/Groningen score was determined based on similar ^18^F-FDG uptake grading in the following areas (10): lumbar interspinous bursae, bilateral sternoclavicular joints, ischial tuberosities, and hips (*n* = 7 sites, max score 14, cut-off 8).

### 2.6 Statistics

Comparison of continuous variables between two independent groups was performed by the Mann Whitney *U* test or student's *t* test. Fisher's exact test or Chi-square test was used for comparison of categorical variables. Cohen's kappa was determined to test agreement between the routine PET scan reports (“PMR” and “not PMR”) vs. outcomes of the ^18^F-FDG-PET/CT scores (“PMR” and “not PMR”. In the latter case, parallel analyses were performed with the “unclear” cases (according to the routine PET scan report) grouped along with either “PMR” or “not PMR” cases. Kappa values were interpreted as follows: <0.2 poor agreement, >0.2 and ≤0.4 fair, >0.4 and ≤0.6 moderate, >0.6 and ≤0.8 good, >0.8 and ≤1 very good. The percentage of cases with uniform agreement was evaluated. Diagnostic accuracy parameters including sensitivity, specificity, diagnostic odds ratio, positive likelihood ratio and negative likelihood ratio were evaluated. Statistical analyses were performed in Graphpad Prism 8, IBM SPSS Statistics 28 and MetaDisc 1.4. *P* values <0.05 were considered statistically significant.

## 3 Results

### 3.1 Patients

In total 68 patients with suspected PMR were included in the study (Table 1), of which 45 (66%) patients were eventually diagnosed with PMR, as established after six months follow-up. While 29 (43%) patients were treatment-naïve, the remaining patients had already received treatment for a putative diagnosis of PMR. Two patients were using glucocorticoid treatment (prednisolone 1 and 5 mg per day, respectively) during the scan, while two patients used a DMARD (in both cases leflunomide). Among the 39 patients who had previously been treated, glucocorticoid therapy had been discontinued within a period of six months prior to the ^18^F-FDG-PET/CT scan in 34 (87%) patients. Five patients had a glucose level ≥7.0 mmol/L during the scan, ranging from 7.4 to 10.4 mmol/L. Alternative diagnosis in non-PMR patients included both inflammatory and non-inflammatory conditions (Table 2).

**Table 1:** Patient characteristics.

Characteristics	All patients (*n* = 68)	PMR (*n* = 45)	Non-PMR (*n* = 23)	*p*-value
Patient characteristics				
Female, no of. patients (%)	42 (62%)	28 (62%)	14 (61%)	0.914
Age, mean (SD) years	67 ± 9	68 ± 9	66 ± 10	0.551
Treatment naïve PMR, no of. patients (%)	29 (43%)	21 (47%)	8 (35%)	0.349
Fulfilling ACR/EULAR criteria for PMR, no. of patients (%)	44 (65%)	35 (78%)	9 (39%)	0.002
CRP at time of scan, median (IQR) mg/L	19 (5–39)	25 (12–44)	8 (1–20)	0.006
ESR at time of scan, median (IQR) mm/hr	40 (18–64)	42 (24–65)	25 (9–63)	0.123
Medication use at time of 18F-FDG-PET/CT				
Using glucocorticoid treatment, no of. patients (%)	2 (3%)	2 (4%)	0 (0%)	0.546
Glucocorticoid treatment dose, median (IQR) mg	3.0 (1.0–5.0)^a^	3.0 (1.0–5.0)^a^	N/A	N/A
Using DMARD, no. of patients (%)	2 (3%)^b^	1 (2%)^b^	1 (4%)^b^	1.000
Cessation of medication within 6 months before 18F-FDG-PET/CT				
Glucocorticoid treatment stopped, no. of patients (%)	34 (50%)	20 (44%)	14 (61%)	0.200
Glucocorticoid treatment stopped, median (IQR) days prior to scan	22 (8–40)	25 (8–52)	15 (7–35)	0.391
DMARD stopped, no. of patients (%)	1 (2%)^c^	0 (0%)	1 (4%)^c^	0.338
DMARD stopped, days prior to scan	90	N/A	90	N/A

Data are shown for 68 patients with suspected PMR. Eventually, 45 patients were diagnosed with PMR after 6 months follow-up. Statistical significance between PMR and non-PMR patients was tested by Manny Whitney *U* test or student's *t* test for continuous variables and the Fisher's exact test or *χ*^2^ test for categorical variables.

^a^ Prednisolone equivalent, data shown for patients using glucocorticoids.

^b^ Leflunomide.

^c^ Methotrexate.

**Table 2:** Alternative diagnosis in patients without PMR.

Alternative diagnosis	Total (*n* = 23)
**Inflammatory rheumatic disease**	**9** **(39.1%)**
Spondyloarthritis	4 (17.4%)
Rheumatoid arthritis	2 (8.7%)
Sjögren's syndrome	1 (4.3%)
ANCA vasculitis	1 (4.3%)
Auto-inflammatory syndrome	1 (4.3%)
**Other condition**	**14** **(60.9%)**
Degenerative condition	3 (13.0%)
Infection related myalgia	2 (8.7%)
Rotator cuff disease	2 (8.7%)
Infection related myalgia and degenerative condition	1 (4.3%)
Osteoarthritis	1 (4.3%)
Medication related myalgia	1 (4.3%)
Fibromyalgia	1 (4.3%)
Bursitis (not PMR related)	1 (4.3%)
Osteoarthritis and fibromyalgia	1 (4.3%)
Tendinopathy	1 (4.3%)

Data are shown for 23 patients receiving an alternative diagnosis, as established after 6 months of follow-up. Bold values represent the total number of patients diagnosed with either a rheumatic inflammatory disease or another condition.

### 3.2 Routine PET scan report

Routine PET scan reports in the 68 patients with suspected PMR were made by 10 nuclear medicine physicians. A total of 54 (79%) reports' conclusions were uniformly interpreted by the three independent readers. Subsequently, a consensus meeting was held among the three readers to resolve the remaining cases where initial agreement was not reached. Eventually, 43 cases were rated as “PMR”, 10 cases as “unclear” and 15 cases as “not-PMR” based on the interpretation of the routine PET scan report. Interpretations as obtained by the consensus meeting were used for subsequent analyses. When grouping “unclear” cases together with those rated as “PMR”, the sensitivity and specificity of the routine PET scan reports were 91% (79–98) and 48% (27–69), respectively (Table 3). Alternatively, when grouping the “unclear” cases together with cases rated as “not-PMR”, the routine PET scan report provided a sensitivity of 82% (68–92) and specificity of 74% (52–90). A sub-analysis of treatment-naïve patients and already treated patient demonstrated better diagnostic accuracy of the routine PET scan reports in the former group (Table 3).

**Table 3:** Diagnostic accuracy of the routine PET scan report.

Patients	Interpretation method	Sensitivity (95% CI)	Specificity (95% CI)	LR+ (95% CI)	LR− (95% CI)	DOR (95% CI)
All patients (*n* = 68)	Routine PET scan report with “inconclusive” cases grouped with “PMR” cases	91 (79–98)	48 (27–69)	1.75 (1.17–2.61)	0.19 (0.07–0.52)	9.40 (2.53–34.92)
	Routine PET scan report with “inconclusive” cases grouped with “not PMR” cases	82 (68–92)	74 (52–90)	3.15 (1.56–6.36)	0.24 (0.12–0.47)	13.10 (3.93–43.69)
Treatment-naïve patients (*n* = 29)	Routine PET scan report with “inconclusive” cases grouped with “PMR” cases	95 (76–100)	63 (25–92)	2.54 (1.03–6.25)	0.08 (0.01–0.56)	33.33 (2.83–392.60)
	Routine PET scan report with “inconclusive” cases grouped with “not PMR” cases	91 (70–99)	88 (47–100)	7.24 (1.15–45.51)	0.11 (0.03–0.42)	66.50 (5.18–853.46)
Already treated patients (*n* = 39)	Routine PET scan report with “inconclusive” cases grouped with “PMR” cases	88 (68–97)	40 (16–68)	1.46 (0.94–2.26)	0.31 (0.09–1.07)	4.67 (0.95–22.90)
	Routine PET scan report with “inconclusive” cases grouped with “not PMR” cases	75 (53–90)	67 (38–88)	2.25 (1.06–4.77)	0.38 (0.17–0.82)	6.00 (1.46–24.73)

Reports were classified as “PMR”, “not PMR” or “inconclusive”. For calculation of the sensitivity and specificity, the “inconclusive” cases were either grouped with the “PMR” cases or the not “PMR” cases. Data are based on 68 patients with suspected PMR.

LR+, positive likelihood ratio; LR−, negative likelihood ratio; DOR, diagnostic odds ratio.

### 3.3 PMR PET scores

The Leuven and Leuven/Groningen Scores, which are based on visual grading of ^18^F-FDG uptake at predefined anatomic sites, were higher in patients with PMR than those without PMR (Table 4). At the predefined cutoff points, the Leuven Score provided a sensitivity of 80% (65–90) and specificity of 74% (52–90) for a diagnosis of PMR when analysing the entire cohort, and the Leuven/Groningen Score a sensitivity of 82% (68–92) and specificity of 74% (52–90) (Table 5). While these PMR PET scores showed similar specificity in treatment-naïve and already treated patients, the sensitivity was substantially higher in the treatment-naïve group. A sub-analysis of already treated patients suggested that the sensitivity of the PMR PET scores was especially low in those that had still been using glucocorticoid treatment up to 3 week prior to the scan (Table 6). The sensitivity of the PMR PET scores was better in patients discontinuing glucocorticoid treatment for ≥3 weeks prior to the scan.

**Table 4:** PMR PET scores in patients with suspected PMR.

Patients	Patient characteristics and [18F]FDG-PET/CT findings			
		PMR	Non-PMR	*p*-value
All patients (*n* = 68)	No. of patients	45	23	
	Leuven score, median (IQR)	19.0 (16.0–21.0)	7.0 (3.0–18.0)	<0.001
	Leuven positive, no. of patients (%)	36 (80.0%)	6 (26.1%)	<0.001
	Leuven/Groningen score, median (IQR)	11.0 (8.5–12.5)	3.0 (1.0–10.0)	<0.001
	Leuven/Groningen positive, no. of patients (%)	37 (82.2%)	6 (26.1%)	<0.001
Treatment-naïve patients (*n* = 29)	No. of patients	21	8	
	Leuven score, median (IQR)	20.0 (19.0–22.5)	4.0 (1.8–17.0)	0.007
	Leuven positive, no. of patients (%)	20 (95.2%)	2 (25.0%)	<0.001
	Leuven/Groningen score, median (IQR)	12.0 (11.5–13.5)	1.0 (0.0–11.5)	0.021
	Leuven/Groningen positive, no. of patients (%)	20 (95.2%)	2 (25.0%)	<0.001
Already treated patients (*n* = 39)	No. of patients	24	15	
	Leuven score, median (IQR)	17.0 (11.0–19.8)	10.0 (3.0–18.0)	0.026
	Leuven positive, no. of patients (%)	16 (66.7%)	4 (26.7%)	0.015
	Leuven/Groningen score, median (IQR)	10.0 (7.0–11.0)	5.0 (2.0–10.0)	0.038
	Leuven/Groningen positive, no. of patients (%)	17 (70.8%)	4 (26.7%)	0.007

Data are shown for 68 patients with suspected PMR. Predefined cutoff values were 16 for the Leuven Score and 8 for Leuven/Groningen Score. Statistical significance was tested using the Mann–Whitney *U* test for continuous variables and the *χ*^2^ test or Fisher's exact test for categorical variables.

**Table 5:** Diagnostic accuracy of the PMR PET scores.

Patients	Interpretation method	Sensitivity (95% CI)	Specificity (95% CI)	LR+ (95% CI)	LR− (95% CI)	DOR (95% CI)
All patients (*n* = 68)	Leuven Score	80 (65–90)	74 (52–90)	3.07 (1.52–6.20)	0.27 (0.14–0.51)	11.33 (3.47–37.00)
	Leuven/Groningen Score	82 (68–92)	74 (52–90)	3.15 (1.56–6.36)	0.24 (0.12–0.47)	13.10 (3.93–43.69)
Treatment-naïve patients (*n* = 29)	Leuven Score	95 (76–100)	75 (35–97)	3.81 (1.14–12.70)	0.06 (0.01–0.49)	60.00 (4.60–782.37)
	Leuven/Groningen Score	95 (76–100)	75 (35–97)	3.81 (1.14–12.70)	0.06 (0.01–0.49)	60.00 (4.60–782.37)
Already treated patients (*n* = 39)	Leuven Score	67 (45–84)	73 (45–92)	2.50 (1.03–6.06)	0.46 (0.24–0.86)	5.50 (1.32–22.86)
	Leuven/Groningen Score	71 (49–87)	73 (45–92)	2.66 (1.10–6.39)	0.40 (0.20–0.80)	6.68 (1.58–28.29)

Data are based on 68 patients with suspected PMR.

LR+, positive likelihood ratio; LR−, negative likelihood ratio; DOR, diagnostic odds ratio.

**Table 6:** Relationship between discontinuation of glucocorticoid treatment and sensitivity of the PMR PET scores.

PMR PET score	Glucocorticoid treatment discontinued <3 weeks prior to the ^18^F-FDG-PET/CT scan (*n* = 8)	Glucocorticoid treatment discontinued ≥3 weeks prior to the ^18^F-FDG-PET/CT scan (*n* = 14)
Leuven Score, sensitivity (95% CI)	50 (16–84)	79 (49–95)
Leuven/Groningen Score, sensitivity (95% CI)	63 (25–92)	79 (49–95)

Data are shown for 22 patients with PMR that had already been treated at time of referral, and who were able to discontinue treatment prior to the ^18^F-FDG-PET/CT scan. In 8 patients, glucocorticoid treatment had been discontinued within 3 weeks prior to the scan. In the remaining 14 patients, glucocorticoid treatment had been stopped ≥3 weeks prior to the scan. Two additional patients with already treated PMR were unable to fully taper the glucocorticoid treatment before the scan. These two patients were using prednisolone 1 and 5 mg per day, respectively, during the scan. In one of the latter patients, the Leuven and Leuven/Groningen Scores were positive. Due to the low number, data for these two patients are not shown in the table.

### 3.4 Comparison between routine PET report and PMR PET scores

**Figure 1:**
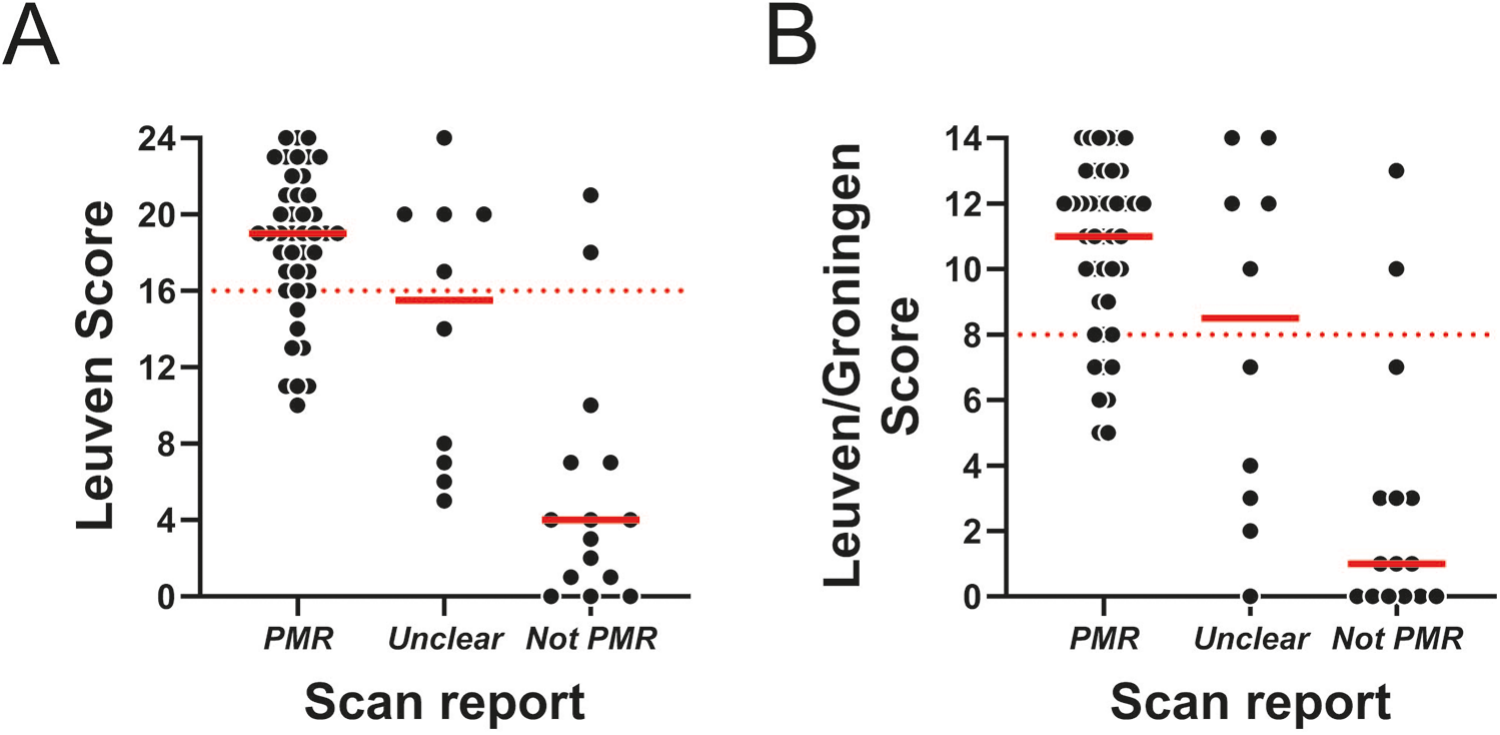
PMR PET scores in relation to routine PET scan report conclusion. **(A)** Leuven Score and **(B)** Leuven/Groningen Score in patients with a positive scan (*n* = 43), unclear scan (*n* = 10) or negative scan (*n* = 15) according to the routine report conclusion. The dashed red line indicates the cutoff for each PMR PET score.

Next, we assessed the relationship between the routine report findings and the PMR PET scores. As expected, the scores were highest when routine PET scan reports were rated as “PMR”, and lowest for reports rated as “non-PMR” (Figure 1). However, the variation in individual scores was substantial, with some cases with low PMR PET score being labelled as “PMR” by the routine PET scan report, and vice versa. Cohen's kappa indicated moderate agreement between the scan report and respective PMR PET scores in the entire cohort, irrespective of whether the “unclear” group was grouped along with scans rated as “PMR” or “not-PMR” (Table 7). When separately analysing treatment-naïve and already treated patients, Cohen's kappa was indicative of good and fair/moderate agreement, respectively. Overall, the agreement was best, when fully excluding the “unclear” group from the analysis, which suggested that prominent disagreement was present in this particular group.

**Table 7:** Agreement between routine PET scan report and the PMR PET scores.

Patients	Routine PET scan reports	Leuven score		Leuven/Groningen score	
		Cohen's kappa	Percent agreement	Cohen's kappa	Percent agreement
All patients (*n* = 68)	Routine PET scan reports with “unclear” cases grouped with “PMR” cases	0.49	78%	0.52	79%
	Routine PET scan reports with “unclear” cases grouped with “not PMR” cases	0.53	78%	0.56	79%
	Routine PET scan reports with “unclear” cases excluded from the analysis	0.60	83%	0.64	84%
Treatment-naïve patients (*n* = 29)	Routine PET scan reports with “unclear” cases grouped with “PMR” cases	0.70	90%	0.70	90%
	Routine PET scan reports with “unclear” cases grouped with “not PMR” cases	0.66	86%	0.66	86%
	Routine PET scan reports with “unclear” cases excluded from the analysis	0.78	92%	0.78	92%
Already treated patients (*n* = 39)	Routine PET scan reports with “unclear” cases grouped with “PMR” cases	0.38	69%	0.41	72%
	Routine PET scan reports with “unclear” cases grouped with “not PMR” cases	0.43	72%	0.48	74%
	Routine PET scan reports with “unclear” cases excluded from the analysis	0.49	75%	0.54	78%

Agreement between routine PET scan report and the Leuven Score or Leuven/Groningen Score regarding a “positive” or “negative” result for PMR. Data are based on 68 patients with suspected PMR. Routine PET scan reports were classified as “PMR” (*n* = 43), “unclear” (*n* = 10) or “not PMR” (*n* = 15). Kappa values were interpreted as follows: <0.2 poor agreement, >0.2 and ≤0.4 fair, >0.4 and ≤0.6 moderate, >0.6 and ≤0.8 good, >0.8 and ≤1 very good.

Finally, we examined whether PMR PET scores could be particularly helpful in patients with an “unclear” scan report conclusion (Table 8). The PMR PET scores correctly classified 7 (70%) of these “unclear” cases as “PMR” or “non-PMR”. In one case, high PMR PET scores were observed, but the widespread inflammation was related to a spondyloarthritis, including sacroiliitis, that was first noted on the ^18^F-FDG-PET/CT. Another patient with high PMR PET scores was diagnosed with infection-related myalgia, without convincing evidence for PMR regarding symptoms, laboratory findings and shoulder/hip ultrasonography. The third patient was diagnosed with PMR, despite limited ^18^F-FDG uptake on PET/CT, which was attributed to low dose glucocorticoid use during the scan.

**Table 8:** Routine PET scan reports interpreted as unclear by the three raters.

#	Routine PET scan report conclusions	Leuven score	Leuven/Groningen score	Diagnosis
1	Could be early PMR.	**17**	**10**	PMR
2	Synovitis of the AC joint and glenohumeral joint bilaterally to a mild degree, possibly reactive to degenerative changes. However, more pronounced synovitis in both hip joints and dorsally of the medial compartment of both knees, as well as tendinopathy in the pelvis. The imaging could be consistent with PMR.	**20**	**12**	PMR
3	Image of extensive polyarthritis. Image consistent with PMR. Bilateral extensive fasciitis lateralis and sacroiliitis.	**24**	**14**	Not-PMR [spondyloarthritis]
4	Light uptake around the shoulder joints, hip joints (especially the greater trochanter bilaterally), and lumbar spinous processes. In terms of activity, no typical image of PMR, although it cannot be completely ruled out given the configuration.	5	0	PMR^a^
5	FDG accumulation around the shoulder and hip joints, ischial bones, as well as accumulation in the soft tissues volar to the carpals. Interpretation: The image could be consistent with PMR, but it is not entirely typical for that. Differential diagnosis could also include polyarthritis.	**20**	**12**	PMR
6	The image could be consistent with moderately active PMR.	8	4	Not PMR [osteoarthritis and fibromyalgia]
7	Slightly increased uptake in the soft tissues around the shoulder joints and the wrist joints. The image could be consistent with a mild form of PMR, differential diagnosis includes degeneration.	7	3	Not PMR [tendinopathy]
8	The image could be consistent with PMR activity.	**20**	**14**	Not PMR [infection related myalgia and degenerative condition]
9	Uptake pattern and locations consistent with PMR, although minimal uptake, so no evidently active PMR.	14	7	Not PMR [spondyloarthritis]
10	PMR based on interspinal FDG accumulation at the lumbar level. No other indications for an explanation of the symptoms.	6	2	Not PMR [degenerative condition]

Conclusions of the ten unclear scan reports are provided, together with the PMR PET scores and eventual diagnosis after 6 months follow-up. Leuven and Leuven/Groningen Scores in bold/underlined are considered positive for PMR. Alternative diagnosis in non-PMR patients is shown between [.].

^a^ Scan performed with 5 mg prednisolone.

## 4 Discussion

In this study, we compared the diagnostic accuracy and agreement between PMR PET scores and routine PET scan reports performed in patients witch suspected PMR. Our study highlights the need for unambiguous reporting of findings by nuclear medicine specialists. Nevertheless, it was reassuring that the diagnostic accuracy of the routine PET scan reports were comparable to that of the PMR PET scores. Additionally, there was good agreement in cases where the nuclear medicine physician could make a clear conclusion (“PMR” vs. “not PMR”). PMR PET scores could be helpful for unclear cases in which the nuclear medicine physician is not able to make a firm conclusion. As such, PMR PET scores provide a more clear and consistent definition for a “positive” scan for PMR.

Clear reporting of ^18^F-FDG-PET/CT findings is important, as ambiguity might impact the interpretation by the physician requesting the scan and subsequent treatment decisions. We observed that in 21% of reports, no agreement was obtained between the readers, when assessing the scan reports' conclusions. Ideally, the reports provide a clear interpretation of the findings, thereby supporting the conclusions of the scan (15). The EANM procedural recommendations for PMR and large vessel vasculitis suggest to examine typical FDG uptake patterns at various musculoskeletal sites according to a 4 point scale grading system (16). In practice, such grading is often not explicitly reported by nuclear medicine physicians. More importantly, no specific guidance on when the FDG uptake is typical enough to rate the scan as “positive” for PMR is given. This might explain why results according to the routine PET scan report and PMR PET scores were discrepant in up to 22% of cases, including cases with limited musculoskeletal ^18^F-FDG uptake being rated as “PMR” in the routine PET scan report.

In order to standardize the interpretation and reporting of ^18^F-FDG-PET/CT, various PMR PET scores and algorithms have been introduced (9–12). The Leuven Score is the best validated score, with its concise version the Leuven/Groningen performing equally well in different patient cohorts (9, 10, 13, 14). In accordance with prior studies, both PMR PET scores showed good sensitivity and specificity for PMR. Recently, the EANM guideline for infection and inflammation recommended the use of the PMR PET scores examined in the current study (17). While these scores may provide a clear definition on whether a scan is “positive” for PMR, the scores cannot be considered absolute evidence for the diagnosis. Indeed it might be difficult for PMR PET scores to distinguish patients with PMR from patients with spondylarthritis, rheumatoid arthritis or reactive inflammatory conditions, if prominent shoulder and hip involvement is present (8, 10). Clinicians should be alert for atypical symptoms, such as prominent arthritis with erosions, which may suggest an alternative rheumatic inflammatory condition that mimics PMR on an ^18^F-FDG-PET/CT scan. Similarly, nuclear medicine specialists should be cautious in diagnosing PMR when there is asymmetric, capsular ^18^F-FDG uptake at large joints or when uptake is observed at the sacroiliac and costovertebral joints.

Despite the discrepant results according to the PMR PET scores and the routine PET scan report in part of patients, their overall diagnostic accuracy was comparable. Although this suggests that neither assessment method is perfect, a diagnostic bias might have occurred in our data as the routine PET scan reports, but not the PMR PET scores, were known to the clinicians making the final diagnosis. This could potentially lead to overestimation of the routine PET scan reports' diagnostic accuracy, making the case for applying PMR PET scores even stronger. Similarly, Nielsen et al. also observed comparable sensitivity and specificity of conventional scan analysis and the Leuven Score in their diagnostic PMR cohort (8). In the latter study, scans were rated as either “PMR” or “not PMR” by conventional analysis. In contrast, the current study also allowed for scans to be rated as “unclear,” better reflecting reports' outcomes seen in daily clinical practice. In 8/10 patients with unclear reports, the PMR PET scores were able to discriminate correctly between patients with a rheumatic inflammatory condition (i.e., 7 cases of PMR, 1 spondyloarthritis with PMR-like presentation) and the non-inflammatory conditions not requiring immunosuppressive therapy. This suggests that the PMR PET scores could perhaps be most useful for cases in which the nuclear medicine physician is uncertain.

Our study cohort was heterogeneous, including both treatment-naïve patients and already treated patients. Since diagnostic imaging is not routinely performed for PMR, already treated patients were often referred due to diagnostic uncertainty following a refractory or relapsing disease course despite treatment. Prior studies have shown that the diagnostic accuracy of ^18^F-FDG-PET/CT scan is significantly lower in patients receiving active treatment (7, 14, 17). To minimize this effect, we aimed to taper therapy in all patients who were referred on treatment. Despite complete glucocorticoid tapering in nearly all patients, the diagnostic accuracy and agreement of routine PET scan reports and PMR PET scores remained markedly lower in already treated patients compared to treatment-naïve patients. This difference might partly reflect the selection of diagnostically challenging cases in the already treated group. Additionally, it could indicate sustained metabolic effects of glucocorticoid therapy that do not resolve promptly after tapering. This aligns with findings from a prospective study in which ^18^F-FDG-PET/CT scans were performed before glucocorticoid treatment, after 8 weeks of therapy, and at week 10 following a 1-week discontinuation (8). In that study, the sensitivity of the Leuven Score declined from 86% at diagnosis to 36% at week 8, and only partially recovered to 66% at week 10. Consequently, the EANM guideline recommends withholding glucocorticoid treatment for at least 2 weeks before scanning, if clinically feasible (18). Bearing in mind the small number of patients in our cross-sectional sub-analysis, our data suggest that glucocorticoid treatment may need to be discontinued for at least 3 weeks prior to the scan. In the latter case, PMR PET scores reached a sensitivity of 79%, whereas a sensitivity 50%–63% was observed if glucocorticoid treatment had been withdrawn for a shorter period of time. While further validation of the latter findings is required, ^18^F-FDG-PET/CT for suspected PMR is ideally performed before initiating treatment to ensure optimal diagnostic accuracy, although costs and practical constraints may limit this approach.

Our study has some additional limitations. The study was performed retrospectively. However, standardized data collection was performed for all patients with suspected PMR in our centre during the inclusion period. PET scan reports were made by ten different nuclear medicine physicians, although this reflects standard clinical practice. The PMR PET scores were established by a single expert reader, and no interrater reliability was performed. However, prior reports have revealed excellent interrater reliability of the scores (8, 13). Furthermore, selection bias might have occurred with only the most difficult cases undergoing ^18^F-FDG-PET/CT. This might have led to underestimation of the diagnostic accuracy. As mentioned, diagnostic bias might have occurred as the final clinical diagnosis was established with knowledge of the routine PET scan report. However, this reflects the absence of true gold standard for PMR.

In conclusion, our study suggests that application of PMR PET scores could be useful for standardized interpretation and reporting of the ^18^F-FDG-PET/CT results in patients with suspected PMR, especially in cases when the nuclear medicine physician is uncertain.

## Data Availability

The original contributions presented in the study are included in the article, further inquiries can be directed to the corresponding author.

## References

[1] Tomelleri A van der Geest KSM Khurshid MA Sebastian A Coath F Robbins D Disease stratification in GCA and PMR: state of the art and future perspectives. Nat Rev Rheumatol. (2023) 19:446–59. 10.1038/s41584-023-00976-8 37308659

[2] Spiera RF Unizony S Warrington KJ Sloane J Giannelou A Nivens MC Sarilumab for relapse of polymyalgia rheumatica during glucocorticoid taper. N Engl J Med. (2023) 389:1263–72. 10.1056/NEJMoa2303452 37792612

[3] Bonelli M Radner H Kerschbaumer A Mrak D Durechova M Stieger J Tocilizumab in patients with new onset polymyalgia rheumatica (PMR-SPARE): a phase 2/3 randomised controlled trial. Ann Rheum Dis. (2022) 81:838–44. 10.1136/annrheumdis-2021-221126 35210264

[4] Devauchelle-Pensec V Carvajal-Alegria G Dernis E Richez C Truchetet M Wendling D Effect of tocilizumab on disease activity in patients with active polymyalgia rheumatica receiving glucocorticoid therapy: a randomized clinical trial. JAMA. (2022) 328:1053–62. 10.1001/jama.2022.15459 36125471 PMC12285571

[5] Mackie SL Koduri G Hill CL Wakefield RJ Hutchings A Loy C Accuracy of musculoskeletal imaging for the diagnosis of polymyalgia rheumatica: systematic review. RMD Open. (2015) 1:e000100–000100. eCollection 2015. 10.1136/rmdopen-2015-000100 26535139 PMC4623371

[6] Slart RHJA Nienhuis PH Glaudemans AWJM Brouwer E Gheysens O Kornelis Svd. Role of ^18^F-FDG PET/CT in large vessel vasculitis and polymyalgia rheumatica. J Nucl Med. (2023) 64:515. 10.2967/jnumed.122.265016 37011940

[7] van der Geest KSM Treglia G Glaudemans AWJM Brouwer E Jamar F Slart RHJA Diagnostic value of [18F]FDG-PET/CT in polymyalgia rheumatica: a systematic review and meta-analysis. Eur J Nucl Med Mol Imaging. (2021) 48:1876–89. 10.1007/s00259-020-05162-6 33372248 PMC8113217

[8] Nielsen AW Hansen IT Nielsen BD Kjær SG Blegvad-Nissen J Rewers K The effect of prednisolone and a short-term prednisolone discontinuation for the diagnostic accuracy of FDG-PET/CT in polymyalgia rheumatica—a prospective study of 101 patients. Eur J Nucl Med Mol Imaging. (2024) 51:2614–24. 10.1007/s00259-024-06697-8 38563881 PMC11224098

[9] Henckaerts L Gheysens O Vanderschueren S Goffin K Blockmans D. Use of 18F-fluorodeoxyglucose positron emission tomography in the diagnosis of polymyalgia rheumatica-A prospective study of 99 patients. Rheumatology (Oxford). (2018) 57:1908–16. 10.1093/rheumatology/kex376 29136209

[10] van der Geest KSM van Sleen Y Nienhuis P Sandovici M Westerdijk N Glaudemans AWJM Comparison and validation of FDG-PET/CT scores for polymyalgia rheumatica. Rheumatology (Oxford). (2022) 61(3):1072–82. 10.1093/rheumatology/keab483 34117743 PMC8889307

[11] Owen CE Poon AMT Yang V McMaster C Lee ST Liew DFL Abnormalities at three musculoskeletal sites on whole-body positron emission tomography/computed tomography can diagnose polymyalgia rheumatica with high sensitivity and specificity. Eur J Nucl Med Mol Imaging. (2020) 47:2461–8. 10.1007/s00259-020-04731-z 32090280

[12] Sondag M Guillot X Verhoeven F Blagosklonov O Prati C Boulahdour H Utility of 18F-fluoro-dexoxyglucose positron emission tomography for the diagnosis of polymyalgia rheumatica: a controlled study. Rheumatology (Oxford). (2016) 55:1452–7. 10.1093/rheumatology/kew202 27107429

[13] Moreel L Boeckxstaens L Betrains A Van Hemelen M Vanderschueren S Van Laere K Diagnostic accuracy and validation of 18F-fluorodeoxyglucose positron emission tomography scores in a large cohort of patients with polymyalgia rheumatica. Front Med (Lausanne). (2022) 9:1026944. 10.3389/fmed.2022.1026944 36213649 PMC9533121

[14] Brinth LS Hansen A Jensen DV Madsen OR Broholm R Krakauer M. Diagnostic value of composite and simplified FDG-PET/CT scores in polymyalgia rheumatica and the influence of recent glucocorticoid treatment-A retrospective diagnostic cohort study. Diagnostics (Basel). (2023) 13:514. 10.3390/diagnostics13030514 36766618 PMC9914179

[15] Niederkohr RD Greenspan BS Prior JO Schöder H Seltzer MA Zukotynski KA Reporting guidance for oncologic 18F-FDG PET/CT imaging. J Nucl Med. (2013) 54:756–61. 10.2967/jnumed.112.112177 23575994

[16] Slart RHJA, Writing group, Reviewer group, Members of EANM Cardiovascular, Members of EANM Infection & Inflammation, Members of Committees SC, et al. FDG-PET/CT(A) imaging in large vessel vasculitis and polymyalgia rheumatica: joint procedural recommendation of the EANM, SNMMI, and the PET Interest Group (PIG), and endorsed by the ASNC. Eur J Nucl Med Mol Imaging. (2018) 45:1250–69. 10.1007/s00259-018-3973-8 29637252 PMC5954002

[17] Casadepax-Soulet C Benali K Crestani B Piekarski E Mahida B Ebstein E Fluorine-18 fluorodeoxyglucose positron emission tomography/computed tomography in polymyalgia rheumatica: an observational study. Clin Exp Rheumatol. (2023) 41:1456–62. 10.55563/clinexprheumatol/kqyki5 36533978

[18] Abikhzer G Treglia G Pelletier-Galarneau M Buscombe J Chiti A Dibble EH EANM/SNMMI guideline/procedure standard for [^18^F]FDG hybrid PET use in infection and inflammation in adults v2.0. Eur J Nucl Med Mol Imaging. (2025) 52:510–38. 10.1007/s00259-024-06915-3 39387894 PMC11732780

